# Efficient discrimination of transplutonium actinides by *in vivo* models†

**DOI:** 10.1039/d0sc06610a

**Published:** 2021-03-10

**Authors:** Roger M. Pallares, Dahlia D. An, Gauthier J.-P. Deblonde, Birgitta Kullgren, Stacey S. Gauny, Erin E. Jarvis, Rebecca J. Abergel

**Affiliations:** Chemical Sciences Division, Lawrence Berkeley National Laboratory Berkeley CA 94720 USA abergel@berkeley.edu; Glenn T. Seaborg Institute, Physical and Life Sciences, Lawrence Livermore National Laboratory Livermore CA 94550 USA; Department of Nuclear Engineering, University of California Berkeley CA 94720 USA

## Abstract

Transplutonium actinides are among the heaviest elements whose macroscale chemical properties can be experimentally tested. Being scarce and hazardous, their chemistry is rather unexplored, and they have traditionally been considered a rather homogeneous group, with most of their characteristics extrapolated from lanthanide surrogates. Newly emerged applications for these elements, combined with their persistent presence in nuclear waste, however, call for a better understanding of their behavior in complex living systems. In this work, we explored the biodistribution and excretion profiles of four transplutonium actinides (^248^Cm, ^249^Bk, ^249^Cf and ^253^Es) in a small animal model, and evaluated their *in vivo* sequestration and decorporation by two therapeutic chelators, diethylenetriamine pentaacetic acid and 3,4,3-LI(1,2-HOPO)*.* Notably, the organ deposition patterns of those transplutonium actinides were element-dependent, particularly in the liver and skeleton, where lower atomic number radionuclides showed up to 7-fold larger liver/skeleton accumulation ratios. Nevertheless, the metal content in multiple organs was significantly decreased for all tested actinides, particularly in the liver, after administering the therapeutic agent 3,4,3-LI(1,2-HOPO) post-contamination. Lastly, the systematic comparison of the radionuclide biodistributions showed discernibly element-dependent organ depositions, which may provide insights into design rules for new bio-inspired chelating systems with high sequestration and separation performance.

## Abstract 2

Transplutonium actinides are among the heaviest elements whose macroscale chemical properties can be experimentally tested.

## Graphical abstract

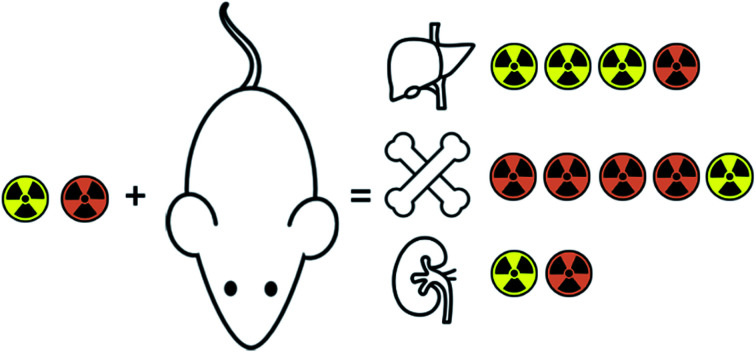

## 1. Introduction

The transplutonium actinides Cm, Bk, Cf and Es are among the heaviest elements whose macroscale chemistry can be experimentally tested.^1,2^ The physical and chemical properties of these radionuclides, however, have not been deeply characterized due to their scarcity, radioactivity, and challenging separations, which are among the most difficult ones within the periodic table.^3^ Nevertheless, these elements are present in various human-driven activities, such as the generation of radioisotope thermoelectric generators,^1,4^ neutron activation sources,^5^ targets for super heavy element discovery,^6^ and nuclear waste.^7^ Past uncontrolled releases of radionuclides to the environment, either accidental or intentional, have demonstrated the need to better understand their behavior *in vivo*, including biodistribution and excretion, as well as to develop decorporation strategies to minimize adverse health effects in humans.^8,9^ The biological outcomes from radiological contamination include both acute and chronic disorders, and their severity depends on multiple factors, such as quantity and duration of exposure.^10^ Among the different types of radiological exposures, internal contamination is particularly dangerous since radionuclides can be deposited in tissues, producing long term radiological poisoning.^11^ Over the last few decades, several chelating molecules have been developed to treat internal actinide contamination by forming highly stable complexes with the metals and enhancing their excretion.^12,13^ For instance, the U.S. Food and Drug Administration (FDA) approved calcium and zinc salts of diethylenetriamine pentaacetic acid (Ca-DTPA and Zn-DTPA) to treat internal contamination with Pu, Am and Cm.^14^ Even though DTPA is the first drug approved to treat this type of radiological contamination, its therapeutic performance is hampered by multiple factors, including (1) a need to administer it in large quantities,^15^ (2) its competition with biological ligands (*e.g.* transferrin and albumin) for the binding of the metal,^16^ and (3) its inability to remove radionuclides deposited in organs.^17^

In order to overcome the limitations of DTPA salts as decorporation agents, the octadentate hydroxypyridinone-based chelator 3,4,3-LI(1,2-HOPO) was developed.^18^ This synthetic chelating molecule shows high binding affinity for actinides and lanthanides,^19,20^ selectivity for f-block elements over biologically-relevant cations,^21,22^ formation of excretable complexes with these elements,^23^ and biocompatibility at therapeutic dosages.^23^*In vivo* studies have demonstrated the superior decorporation performance of 3,4,3-LI(1,2-HOPO) for multiple elements, including U(vi), Np(v), and Pu(iv), when compared to DTPA and other chelating agents.^23–25^ As a result, 3,4,3-LI(1,2-HOPO) has been approved for Phase 1 first-in-human clinical studies, as a decorporation agent for actinides.^18^ The aforementioned *in vivo* studies, however, focused on lanthanides and earlier actinides, and recent spectroscopic and separation studies with Cm, Bk and Cf revealed coordination chemistry differences,^19,26,27^ prompting us to question whether such effects could translate to different biodistribution patterns and if 3,4,3-LI(1,2-HOPO) would preserve its decorporation performance amongst heavier radionuclides.

**Fig. 1:**
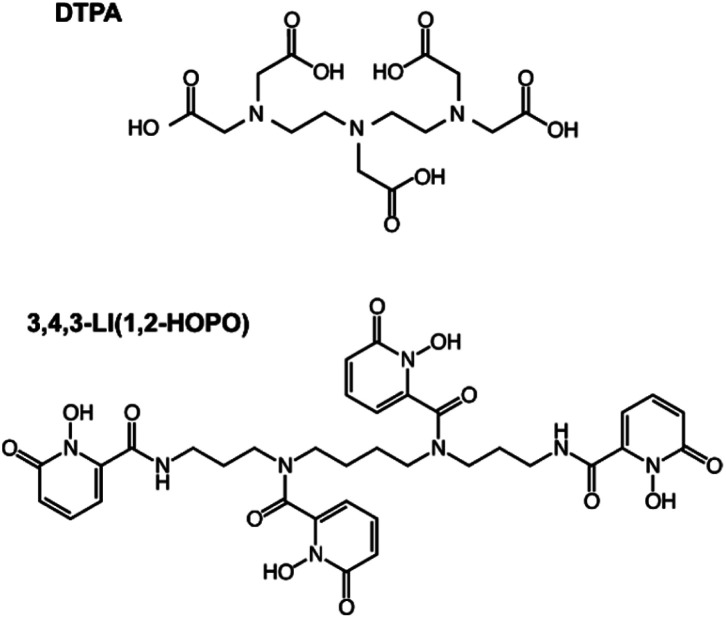
Structures of the chelating agents used in this study. DTPA (top) and 3,4,3-LI(1,2-HOPO) (bottom).

Here we present the internal accumulation and excretion profiles in mice contaminated with ^248^Cm, ^249^Bk, ^249^Cf and ^253^Es. Although the main two deposition regions, skeleton and liver, were the same for the four metals, the total organ retention and evolution over time was element-dependent, with lower atomic number radionuclides showing higher liver and lower skeleton accumulations. Prompt post-contamination decorporation treatments with DTPA and 3,4,3-LI(1,2-HOPO) (Fig. 1) resulted in enhanced radionuclide clearance, with the latter ligand showing better therapeutic performance. Rather unexpectedly, these results also indicate that although transplutonium actinides present common characteristics as a series, their *in vivo* biodistribution and clearance, in the absence and presence of therapeutic chelators, are discernibly element-dependent.

## 2. Results and discussion

### 2.1. Biodistribution and preferential organ deposition

The biodistribution of transplutonium elements was tested in young adult female Swiss-Webster mice. ^248^Cm, ^249^Bk, ^249^Cf, and ^253^Es were chosen for their availability and because their half-lives (3.4 × 10^5^ years, 330 days, 351 years, and 20.5 days, respectively) were long enough to perform the experiments. The radionuclides were injected intravenously as citrate solutions with final administered activities for each actinide ranging between 0.23 and 0.93 kBq per mouse (Table S1†). These activities were high enough to be traced but low enough to avoid acute radiation effects on the animals. ^249^Cf and ^249^Bk were injected together due to their availability in our laboratory. Nevertheless, it is highly unlikely they interfered with each other's biodistribution, considering the large excess of endogenous chelators in blood. While ^249^Cf and ^249^Bk concentrations were in the nanomolar and picomolar range in the bolus solutions (before injection and dilution in the mouse circulatory system), metal-binding proteins, such as hemoglobin, transferrin, and fetuin, are in the micromolar and millimolar range in mouse blood (Table S2†). It is worth noting that the concentrations of the different species affect the binding equilibrium, and we had to use different injected masses for each actinide due to their distinct activities (Table S1†). However, the large excess of binding proteins in blood compared to the injected radioisotopes likely minimized any actinide concentration effect in biodistribution by pulling the equilibrium towards the protein-actinide complexes through Le Chatelier's principle. The mice were euthanized at different time points (from 5 min to 24 h post contamination), and their tissues and excreta were collected and radioanalyzed. For decorporation efficacy tests, two mouse groups received a DTPA or 3,4,3-LI(1,2-HOPO) treatment (30 μmol kg^−1^ per mouse) through intraperitoneal injection 1 h after contamination, an administration time widely used to assess decorporation treatments,^18^ and were euthanized 24 h after actinide exposure. All mice showed steady body weight, and no visible or palpable dermal infections, impaired mobility, or ascites were detected during the study, indicating a lack of acute toxicity.

**Fig. 2:**
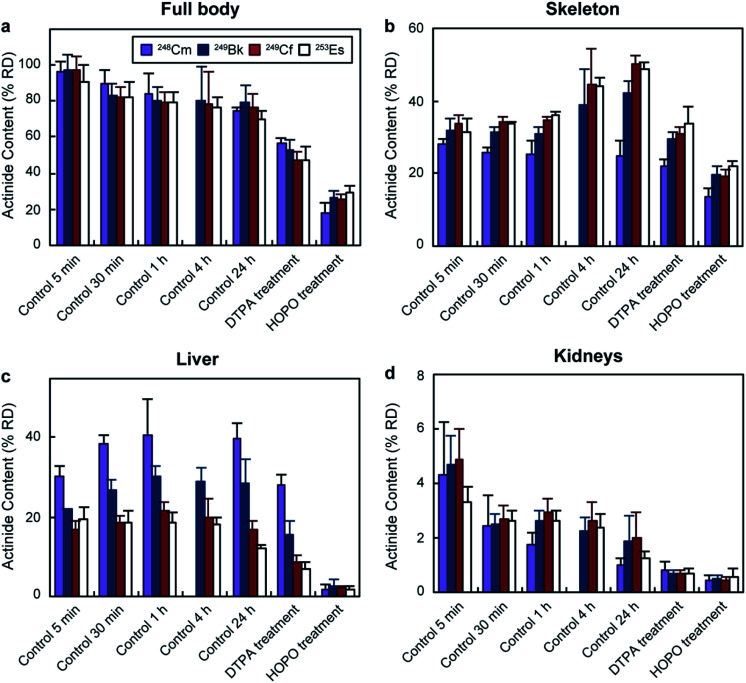
Total actinide body content and distribution following contamination *via* intravenous injection. Results are reported as percentage of recovered dose (% RD, mean ± SD, *n* = 5) recovered from (a) full body, (b) skeleton, (c) liver, and (d) kidneys. HOPO stands for 3,4,3-LI(1,2-HOPO). Full body accounts for all the dose recovered from a mouse but the dose from the excreta.

Fig. 2 shows the total percentage of actinide recovered dose (% RD) and their distribution in selected organs for each group. In the absence of treatment, the total body retention stabilized around 80% RD (Fig. 2a) for all four actinides after 1 h. Although the total body content remained fairly similar from 1 to 24 h, the organ distribution changed over time. One of the main accumulation regions was the skeleton, which showed distinct deposition behaviours for the four isotopes. ^248^Cm accumulation in the bones was steady (between 28 ± 2 and 24 ± 4% RD) over 24 h, while ^249^Bk, ^249^Cf and ^253^Es content increased from around 32% up to 42 ± 3, 50 ± 2, and 49 ± 2% RD, respectively. The different behaviours between the four isotopes were more pronounced in the liver, the organ with the second largest actinide content (Fig. 2c). ^248^Cm and ^249^Bk showed up to 40 ± 4 and 30 ± 3% RD accumulation, respectively, during the experiments, which contrasted with the lower liver deposition observed for ^249^Cf (17 ± 2% RD) and ^253^Es (12 ± 1% RD) after 24 h. The higher liver accumulation by earlier transplutonium actinides is consistent with a past study with trivalent ^241^Am (0.9 kBq per mouse), which showed 49% RD liver content after 24 h.^18^ The experimental protocol used in ref. 18 is the same as that used in our current study. Regarding other organs, kidneys (Fig. 2d) and soft tissues (Fig. S1†) showed smaller actinide content (<5% RD after 24 h), which decreased over time for all tested radionuclides. The preference of transplutonium elements for skeleton and liver is in agreement with other biodistribution studies performed with lighter actinides.^23–25^

**Fig. 3:**
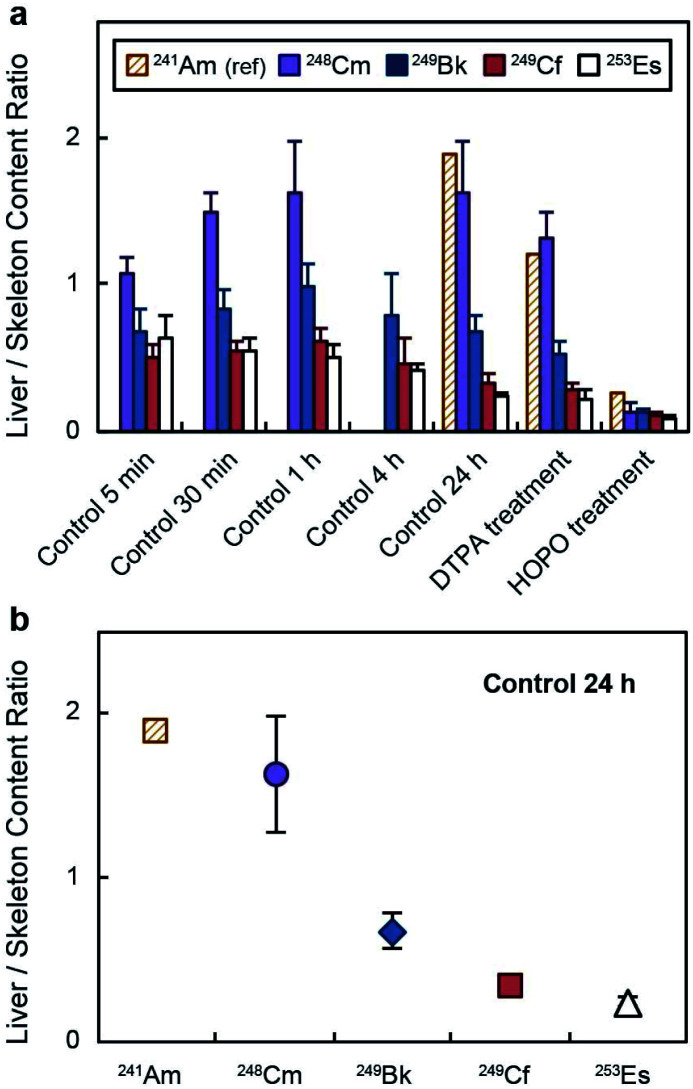
Liver/skeleton actinide deposition ratio. Deposition ratios (mean ± SD, *n* = 5) (a) at different time points and (b) at 24 h post contamination. HOPO stands for 3,4,3-LI(1,2-HOPO). ^241^Am data was obtained from ref. 18, which followed the same experimental protocol as that used in this study. The injected dose of ^241^Am was 0.9 kBq per mouse.

The distinct deposition profiles for the different transplutonium actinides are clearly visible in Fig. 3a, which shows a decrease in the actinide liver/skeleton accumulation ratio with increasing atomic number. These results are consistent with liver and skeleton biodistribution trends across the lanthanide series, which are also element-dependent.^28^ The deposition trend was more pronounced at 24 h post contamination (Fig. 3b), when the lighter transplutonium actinide studied here, ^248^Cm, showed around 7-fold higher liver/skeleton accumulation ratio than ^253^Es. Although mammals are not known to use actinides for essential biochemical processes, f-block elements may compete with Ca^2+^, Fe^3+^, Mg^2+^, and Mn^2+^ for protein metal-binding sites,^29,30^ and the observed differences in organ accumulation ratios likely stem from element discrimination at the molecular level. For instance, one identified mammalian target of lanthanides and actinides after internal contamination is fetuin, a calcium-binding protein that participates in bone metabolism.^31–33^ Moreover, actinides also show high binding affinity for several proteins that participate in the shuttling of calcium to bone tissue.^34^ Even though limited data comparing the interaction between bone-related proteins and trivalent actinides has been published, one study reported Cm^3+^ having higher binding affinity for multiple bone glycoproteins than Am^3+^,^34^ which may explain the larger bone deposition of trivalent actinides with higher atomic number.

Regarding liver accumulation, actinides interact with multiple metal-binding proteins in the liver uptake pathway, including transferrin, ferritin, and calmodulin.^35,36^ Transferrin is reported to be the main protein mediating actinide transport from blood to hepatic cells, and once actinides are internalized, they are transferred to other high molecular weight proteins.^35,36^ A key step in this acquisition pathway is the interaction between the metal–transferrin complex and the cell surface receptor that mediates endocytosis of the complex. For instance, Pu^4+^ shows high affinity for transferrin, but the resulting complexes (Pu^4+^–transferrin) are poorly recognized by the transferrin receptor, and only one isoform containing one Pu^4+^ ion and one Fe^3+^ ion is actively recognized and endocytosed.^37^ Hence, Pu^4+^ is only moderately internalized by hepatic cells compared to other metals.^38^ Thus, the direct affinities between the metals and the transferrin may not be as important on defining the actinide liver uptakes, as the interactions between the metal complexes and the transferrin receptor are. There is not a systematic study that explores the binding between trivalent actinide–transferrin complexes and the receptor. However, a study with lanthanides indicated decreasing affinity of the receptor for the metal–transferrin complexes with increasing atomic number (La^3+^ ∼ Nd^3+^ > Gd^3+^ > Yb^3+^),^39^ which, if trivalent actinides follow the same trend, may explain the larger liver deposition of early transplutonium elements that we observed. It is worth noting that the burden transition from a higher liver accumulation to a higher skeleton accumulation occurs between Cm^3+^ and Bk^3+^ for the actinides (Fig. 3) and between Sm^3+^ and Eu^3+^ for the lanthanides, according to data compiled and reviewed by Leggett *et al.*^28^ If the biochemical processes involved in the transport of these exogenous metals were exclusively driven by ionic interactions, the lanthanide transition would appear around Pm^3+^ (with an ionic radius intermediate between those of Cm^3+^ and Bk^3+^) or the actinide transition would appear around Cf^3+^ (with an ionic radius intermediate between those of Sm^3+^ and Eu^3+^).^40^ This transition, however, could be explained based on differences on covalency between the lanthanide and actinide series. Over the past decade, a combination of experimental and theoretical studies has evidenced increasing covalent character in bonding across the late actinide series, contrasting with the trivalent lanthanides.^41–43^ In addition, small molecular transplutonium complexes have been shown to display strong energy degeneracy-driven covalency, distinct from the more traditional overlap-driven covalency typical of the early actinides.^44–46^ Those are very subtle differences but they could still affect several features, including bond lengths and electron density around the metal center, metal binding kinetics, or metal complex thermodynamic stability and could potentially explain variations in 4f- or 5f-element binding by different biological molecules, such as bone glycoproteins, small molecular ligands circulating in the blood or common metalloproteins.

Lastly, the absorbed dose rates for each radionuclide in the liver and skeleton (the main two deposition regions) were calculated at 24 h to further confirm that the injected doses were low enough to avoid any acute radiation damage. The absorbed dose rates were sub-μGy/s for all actinides (Table S3†), below the dose rates commonly used in mouse experiments.^47^

### 2.2. Decorporation treatments against internal contamination

Mice that were treated with chelating agents received the treatment 1 h after contamination, and were euthanized 24 h post-metal exposure. Administration of DTPA reduced the total retained ^248^Cm, ^249^Bk, ^249^Cf, and ^253^Es to 57 ± 3, 53 ± 6, 47 ± 5, and 48 ± 7% RD, respectively (Fig. 2). The bodily actinide content of the mice treated with 3,4,3-LI(1,2-HOPO) was below 30% RD for all four radionuclides, which corresponded to roughly 3 and 2-fold decreases relative to the control and DTPA-treated groups, respectively. Although actinide deposition in all organs notably decreased after 3,4,3-LI(1,2-HOPO) treatment, the changes in liver activity were the most significant ones, with reductions ranging from 6 to 23-fold compared to the control group. This is of particular interest since liver cancer is one of the main disorders associated with internal radionuclide contamination.^48^ One of the reasons 3,4,3-LI(1,2-HOPO) significantly prevented actinide accumulation in liver despite the 1 h delayed treatment is the chelator's fast biodistribution into this organ, which occurs within 5 min after injection.^49^

### 2.3. Excretion profiles

**Fig. 4:**
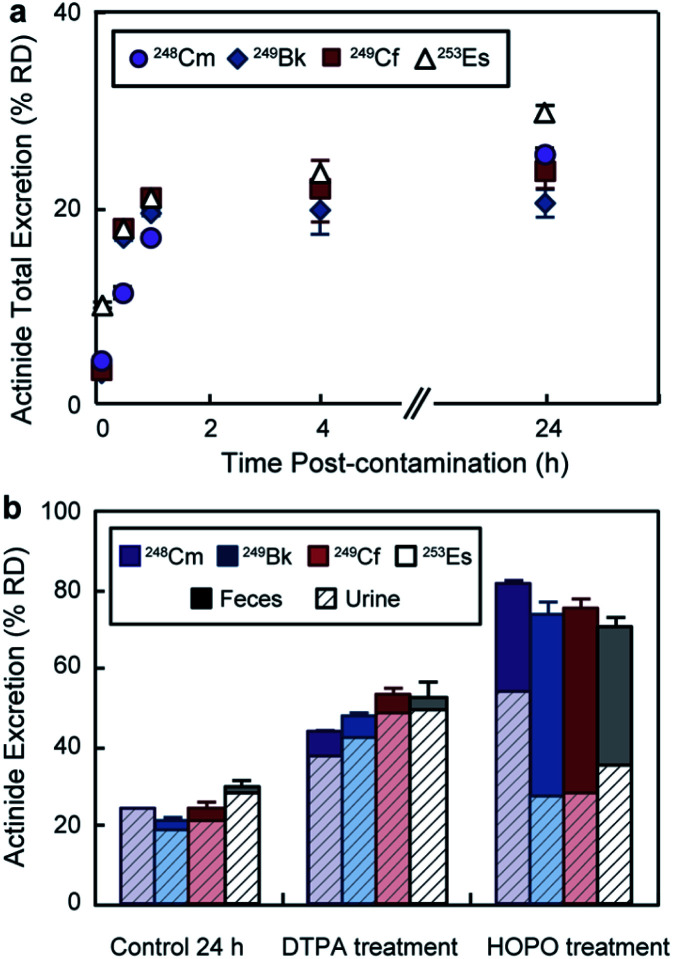
An^3+^ excretion following contamination *via* intravenous injection. (a) Cumulative excretion (urine and feces) of control groups over time. In absence of treatment, the four radionuclides had similar excretion profiles with the largest portion of actinide being excreted within 30 min. (b) Actinide excreted at 24 h post contamination and excretion routes. Feces and urine excretion are displayed as solid and dashed columns, respectively. Results are reported as percentage of recovered dose (% RD, mean ± SD, *n* = 5). HOPO stands for 3,4,3-LI(1,2-HOPO).

In addition to the bioaccumulation studies, the mice excreta were also collected and radioanalyzed. Fig. 4a displays cumulative actinide excretion over time in the absence of treatment. The four metals had similar elimination profiles, where the largest portion of the radionuclides was excreted within the first 30 min post-contamination. For the treatment groups (Fig. 4b), total excretion after 24 h was significantly higher than for the control group and inversely proportional to body accumulation. Treatment with DTPA promoted ∼ 50% RD clearance, which occurred primarily through the urinary pathway. 3,4,3-LI(1,2-HOPO), on the other hand, showed superior excretion rates with over 70% RD for all four actinides, and a significant increased fecal elimination component. The different excretion routes for DTPA and 3,4,3-LI(1,2-HOPO) complexes have been previously observed and explained based on the metal complex physico-chemical properties, including lipophilicity, solubility and ionization constants.^24^ Noteworthy, we observed significant differences and opposite trends in ligand-promoted excretion as a function of the metal, following the order ^248^Cm < ^249^Bk < ^249^Cf ∼ ^253^Es for DTPA and ^253^Es < ^249^Cf ∼ ^249^Bk < ^248^Cm for 3,4,3-LI(1,2-HOPO). The trend seen with DTPA correlates with increasing stability constants for complexes formed from Cm to Es (Table S4†), with higher metal affinities corresponding to higher excretion and decorporation power. In contrast, the effect seen with 3,4,3-LI(1,2-HOPO) is reversed from those observed for DTPA, and could be traced back to the decreasing amount of actinide initially deposited in the liver, when progressing from ^248^Cm to ^253^Es, as the liver is a direct pool for decorporation by 3,4,3-LI(1,2-HOPO), which is not the case for DTPA.

### 2.4. Systematic comparisons of actinide biodistribution profiles

**Fig. 5:**
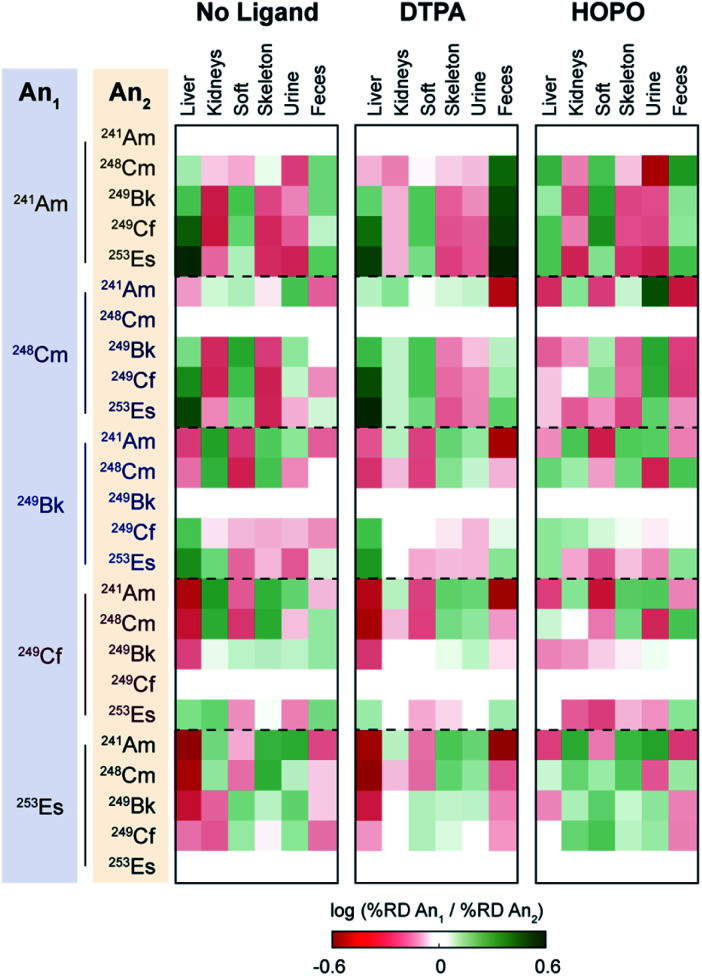
Relative accumulation ratios of actinides in different organs and excreta at 24 h post contamination. The therapeutic ligands were administrated 1 h after contamination. HOPO stands for 3,4,3-LI(1,2-HOPO). ^241^Am data was obtained from ref. 18, which followed the same experimental protocol as that used in this study. The injected dose of ^241^Am was 0.9 kBq per mouse.

To further provide a thorough perspective of the different actinide bioaccumulation profiles as well as their respective variations after chelator administration, we systematically compared deposition ratios of the tested radionuclides 
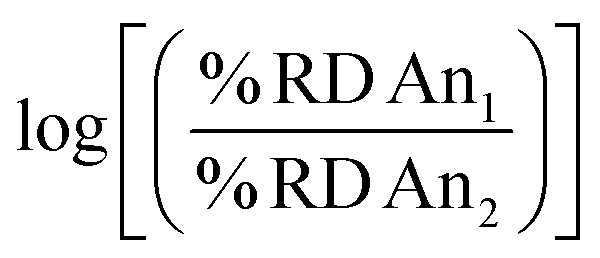
 in each organ and excreta tested, for the control, HOPO treatment, and DTPA treatment groups, 1 h post-contamination (Fig. 5). In this analysis, similar data previously collected and reported on ^241^Am were included.^18^ As mentioned above, lighter transplutonium actinides tended to accumulate to larger extent in the liver compared to heavier ones, which showed higher deposition in the skeleton. This trend is mostly reproduced, albeit sometimes attenuated, after administration of a chelating agent, even though a few features are worth noting. Although DTPA promoted the excretion of actinides primarily through urine, its administration yielded a large increase of ^241^Am expulsion through the feces compared to all heavier radionuclides studied. This is best exemplified by the brighter green “Feces” column on the top centre panel of Fig. 5, as well as the brighter red upper right corners of each of the remaining centre panels. In contrast, administration of 3,4,3-LI(1,2-HOPO) seems to accentuate the differences in excretion between ^241^Am and ^248^Cm, two actinides notoriously difficult to separate: while the urinary (fecal) output of ^241^Am is smaller (larger, respectively) than that of ^248^Cm in control contaminated animals, that difference is much increased after one 3,4,3-LI(1,2-HOPO) chelation treatment, as depicted by the much brighter colours in the upper right corner of the top right panel of Fig. 5, compared with the top left panel. This 3,4,3-LI(1,2-HOPO)-induced excretion change was also concomitant with a reversed relative liver retention pattern. Thus, even though 3,4,3-LI(1,2-HOPO) showed the highest therapeutic performance as decorporating agent, both ligands had substantial effects on changing the relative biodistributions of the tested actinides, which could be of particular interest if one envisioned to exploit these different distribution ratios for purposed element separations in bio-inspired artificial systems. Mostly motivated by the need to devise new efficient strategies for the mining and purification of rare earth metals as critical materials^50^ or for the large-scale extraction of these elements from diluted environments such as seawater,^51^ many biological and biologically-inspired molecules have recently emerged as potentially promising systems for f-element separations. Current state-of-the-art in this field runs the gamut from biopolymers, organic acids, as well as small molecule metallophores, peptides and proteins produced by microbial species.^52^ However, mammalian systems have not yet been explored in that context. The results presented here indicate that the molecular mechanisms involved in the mammalian transport and storage of actinide contaminants could eventually be decrypted, improved, and utilized to discriminate f-block metals. One may even envision the engineering of new bioreactors, such as modified spheroid reservoir bioartificial livers,^53^ that would leverage these mechanisms.

## 3. Conclusions

In summary, we studied the biodistribution and excretion of the heaviest transplutonium elements available, namely ^248^Cm, ^249^Bk, ^249^Cf and ^253^Es, in a live mouse model. Their deposition in organs was observed within the first five minutes after contamination, and skeleton and liver were the main accumulation regions. Despite their similar charge and ionic radius, the distribution of radionuclides was element dependent, where lower atomic number transplutonium elements showed up to 7-fold higher liver/skeleton deposition ratios compared to the heavier tested actinides. Treatment with both DTPA and 3,4,3-LI(1,2-HOPO) 1 h after contamination promoted higher radioisotope excretion, with the latter showing better performance (around 3-fold higher clearance compared to control). Although mice do not use actinides in their metabolism, transplutonium element interaction with endogenous chelators seems to be element dependent, resulting in different *in vivo* behaviour. Such discernible differences in a complex multi-component living system may provide future insights for developing new bio-inspired strategies for efficient sequestration and separation of transplutonium elements.

## Author contributions

D. D. A., G. J.-P. D., B. K., and R. J. A. designed the research. G. J.-P. D. and R. J. A. prepared radioisotope solutions for experimental procedures. D. D. A., B. K., S. S. G., and E. E. J. performed *in vivo* experiments. R. M. P., D. D. A., G. J.-P. D., and R. J. A. analyzed the data. All authors discussed the experimental results and contributed to the manuscript.

## Conflicts of interest

R. J. A. and G. J.-P. D. are listed as inventors on patent applications filed by the Lawrence Berkeley National Laboratory (LBNL) and describing inventions related to the research results presented here. The authors declare no competing financial interest.

## Supplementary Material

SC-012-D0SC06610A-s001
