# Supplementary material for: Efficient discrimination of transplutonium actinides by in vivo models
Source: Chem Sci. 2021 Mar 10;12(14):5295–301. doi: 10.1039/d0sc06610a (PMC8179619; doi:10.1039/d0sc06610a)
Supplement: SC-012-D0SC06610A-s001 [file SC-012-D0SC06610A-s001.pdf]

Electronic Supplementary Material (ESI) for Chemical Science.
This journal is © The Royal Society of Chemistry 2021

Efficient discrimination of transplutonium actinides by *in vivo* models

Roger M. Pallares,[a] Dahlia An,[a] Gauthier J. –P. Deblonde,[a,b] Birgitta Kullgren,[a] Stacey Gauny,[a] Erin E. Jarvis,[a] and Rebecca J. Abergel[a,c,*]

[a]Chemical Sciences Division, Lawrence Berkeley National Laboratory, Berkeley, CA, 94720, USA

[b]Glenn T. Seaborg Institute, Physical and Life Sciences, Lawrence Livermore National Laboratory, Livermore, CA, 94550, USA

[c]Department of Nuclear Engineering, University of California, Berkeley, CA, 94720, USA

*E-mail: abergel@berkeley.edu

Table of Contents

Experimental Section S2

Table S1. Composition of actinide and ligand injected solutions. S4

Table S2. Concentrations of metal-binding proteins in mouse blood, plasma and serum. S5

Figure S1. Soft tissue deposition of different actinides. S6

Table S3. Absorbed dose rates in liver and skeleton at 24 h. S7

Table S4. Stability constants of transplutonium actinides with DTPA. S8

Supporting Information References S9

Experimental Section

Caution: *^{248}Cm, ^{249}Bk, ^{249}Cf, and ^{253}Es are radioactive and should be manipulated only in a specifically designated facility, in accordance with appropriate safety controls. All measurements were undertaken either in controlled facilities and/or using multiple containment procedures.*

General. All chemicals were obtained from commercial suppliers and were used as received. The ligand 3,4,3-LI(1,2-HOPO) was synthesized by Ash Stevens, Inc. (Detroit, MI, USA), following a previously reported procedure, and used as received.[1] DTPA was purchased from Sigma-Aldrich (St. Louis, MO), and formulated as Ca-DTPA using NaOH and $CaCO_3$. Aliquots of acidified stocks of carrier-free ^{248}Cm (95.78% ^{248}Cm, 4.12% ^{246}Cm, 0.06% ^{245}Cm, 0.02% ^{244}Cm/^{247}Cm isotopic distribution by atom percentage) were obtained from Lawrence Berkeley National Laboratory. ^{253}Es was provided by Prof. J. C. Shafer (Department of Chemistry, Colorado School of Mines, Golden, CO). $^{249}BkCl_3$ and $^{249}CfCl_3$ were obtained from the National Isotope Development Center at Oak Ridge National Laboratory (Oak Ridge, TN), and diluted in HCl (0.1 M). Injection solutions (0.2 mL) were prepared containing either ^{253}Es (final activity 0.370 kBq/mouse), a mixture of ^{249}Cf and ^{249}Bk (final activity 0.925 kBq/mouse for each radionuclide, resulting in a total mixture activity of 1.850 kBq/mouse), or ^{248}Cm (0.230 kBq/mouse) in 8 mM sodium citrate and 0.14 M NaCl at pH 4. Chelating agent solutions were prepared such that 30 µmol/kg mouse were contained in 0.5 mL (0.14 M NaCl, with pH adjusted between 7.4 and 8.4 with 1 N NaOH). All solutions were sterilized with 0.22 µm filters prior to administration. All aqueous solutions were prepared using deionized water purified through a Millipore Milli-Q reverse osmosis cartridge system.

Animals and general procedures. All protocols and procedures used in the *in vivo* studies were reviewed and approved by the Institutional Animal Care and Use Committee of Lawrence Berkeley National Laboratory, and were performed in AAALAC accredited facilities, in compliance with guidelines from the Public Health Service's National Institutes of Health Office of Laboratory Animal Welfare. The animals used were young adult (89 days old for contamination experiment) female (31.7 ± 2.1 g) Swiss Webster mice (Simonsen Laboratories, Gilroy, CA). Mice were kept under a 12 h light cycle with controlled temperature (18 – 22 ˚C) and relative humidity (30 – 70 %), and were given food and water *ad libitum*. Each group (five mice per group) was housed together in a plastic stock caged lined with a 0.5 cm layer of highly absorbent low-ash pelleted cellulose bedding (ALPHA-dri®) for separation of urine and feces.

Intravenous (iv) injections into a warmed lateral tail vein, intraperitoneal (ip) injections, and euthanasia were performed under anesthesia. For biodistribution experiments, mice were injected iv with a single dose (0.2 ml) of either ^{249}Bk- and ^{249}Cf-citrate mixture (0.925 kBq each metal), ^{253}Es-citrate (0.370 kBq) or ^{248}Cm-citrate (0.230 kBq) solutions. For a full description of the injection solutions please refer to Table S1. Animals were euthanized at different time points, and their excreta were collected. To probe the decorporation effects of the ligands, mice were first administered the radionuclide solutions iv (0.2 mL), and then injected ip with the ligand treatment 1 h post-contamination (~0.5 mL). The treatment dose volumes were adjusted based on the mouse weight, where 0.5 mL volume corresponded to a 35-g mouse. Injection volumes were consistent with the dose volumes recommended by multiple guidlines.[2, 3] Euthanasia was performed by cervical dislocation over mouse groups' respective cages, excreta expelled at death were collected, and the animals were immediately wrapped in plastic and frozen for later dissection. After partial thawing, liver and kidneys were dissected, and the reminder soft tissue (*e.g.* muscle, fibrous tissue, and fat) was removed. The organs and eviscerated carcasses were handled as individual samples. Feces were separated manually from urine-stained cellulose bedding, and treated as group samples (one feces sample and one urine sample per cage). All samples were dried at 100 °C and dry-ashed at 575 °C. The ashed samples were dissolved in concentrated HNO_3, and the resulting solutions were homogenized in diluted HNO_3 and mixed with Ultima Gold (Perkin Elmer, Shelton, CT, USA). Alpha and beta radioactivity was then determined by liquid scintillation counting on a Perkin Elmer Packard Tri-Carb model B4430. Because the instrument distinguishes between alpha and beta radiation, the ^{249}Cf (α emitter) and ^{249}Bk (β emitter) content of the samples could be discriminated. The experimental results were reported as percent of recovered dose (% RD), which were calculated by adding up all the doses recovered for each mouse (these included activities from organs, skeleton, and excreta). All radiochemical recoveries were greater than 95% injected dose. Urine and feces were counted as group samples, and their values were divided by the number of mice in the cage. Hence, all mice in the same cage had the same urine and feces activity values. However, because their total recovered dose was different (dose in organs, feces and urine), each mouse had different feces and urine % RD. Lastly, for practical purposes, collection of urine and feces at early time points was done by placing the mice in a new cage, and collecting the old urine-stained cellulose bedding and manually picking the feces.

Table S1. Composition of actinide and ligand injection solutions.

Injection	***Actinide***	***Ligand***	***Concentration***	***Activity***	***Injection Volume***	***Injection Route***
1	^{253}Es		7.85 pM	1.85 kBq/mL	0.2 mL	iv
2	^{249}Cf<br>^{249}Bk		123 nM<br>316 pM	4.63 kBq/mL<br>4.63 kBq/mL	0.2 mL	iv
3	^{248}Cm		29.5 μM	1.15 kBq/mL	0.2 mL	iv
4		3,4,3-LI(1,2-HOPO)	30 μmol / kg mouse		0.5 mL / 35-g mouse	ip
5		DTPA	30 μmol / kg mouse		0.5 mL / 35-g mouse	ip

Actinide solutions contained 8 mM sodium citrate.
All solutions contained 0.14 M NaCl.

Table S2. Concentrations of metal-binding proteins in mouse blood, plasma and serum.

Protein	***Concentration (g/L)***	***Concentration (μM)***	***Fluid***	***Reference***
Hemoglobin	150	10400	Blood	4
Transferrin	2	25	Plasma	5
Fetuin (young mice)	1	16.9	Serum	6
Fetuin (adult mice)	0.2	3.4	Serum	6

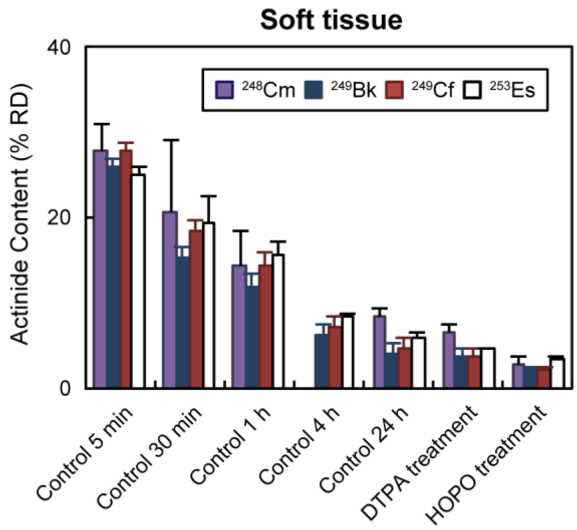

Figure S1. Soft tissue deposition of different actinides.

Table S3. Absorbed dose rates in liver and skeleton at 24 h.

Actinide	***Liver (Gy/s)***	***Skeleton (Gy/s)***
^{248}Cm	$5.2 \pm 0.5 \times 10^{-8}$	$2.1 \pm 0.4 \times 10^{-8}$
^{249}Bk	$3.7 \pm 0.4 \times 10^{-9}$	$3.6 \pm 0.3 \times 10^{-9}$
^{249}Cf	$1.0 \pm 0.1 \times 10^{-7}$	$2.0 \pm 0.1 \times 10^{-7}$
^{253}Es	$3.3 \pm 0.3 \times 10^{-8}$	$8.8 \pm 0.3 \times 10^{-8}$

Table S4. Stability constants of transplutonium actinides with DTPA.

Actinide	*An(DTPA)$^{2-}$*	*Reference*
Cm^{3+}	21.67 ± 0.09 *	7
Bk^{3+}	23.4 ± 0.1 **	8
Cf^{3+}	23.8 ± 0.1 **	8
Es^{3+}	24.04 ± 0.01**	8

*Measured at ionic strength of 1.0 M $NaClO_4$
**Measured at ionic strength of 0.5 M $NaClO_4$

Supporting Information References

1. R. J. Abergel, P. W. Durbin, B. Kullgren, S. N. Ebbe, J. Xu, P. Y. Chang, D. I. Bunin, E. A. Blakely, K. A. Bjornstad, C. J. Rosen, D. K. Shuh and K. N. Raymond, *Health Phys.*, 2010, **99**, 401-407.
2. S. C. Gad, C. B. Spainhour, C. Shoemake, D. R. S. Pallman, A. Stricker-Krongrad, P. A. Downing, R. E. Seals, L. A. Eagle, K. Polhamus and J. Daly, *International Journal of Toxicology*, 2016, **35**, 95-178.
3. R. M. Hull, *Human & Experimental Toxicology*, 1995, **14**, 305-307.
4. B. M. Raabe, J. E. Artwohl, J. E. Purcell, J. Lovaglio and J. D. Fortman, *J. Am. Assoc. Lab. Anim. Sci.*, 2011, **50**, 680-685.
5. E. A. Malecki, A. G. Devenyi, J. L. Beard and J. R. Connor, *BioMetals*, 1998, **11**, 265-276.
6. W. Jahnen-Dechent, T. Schinke, A. Trindl, W. Müller-Esterl, F. Sablitzky, S. Kaiser and M. Blessing, *J. Biol. Chem.*, 1997, **272**, 31496-31503.
7. G. Tian, Z. Zhang, L. R. Martin and L. Rao, *Inorg. Chem.*, 2015, **54**, 1232-1239.
8. M. P. Kelley, N. P. Bessen, J. Su, M. Urban, S. I. Sinkov, G. J. Lumetta, E. R. Batista, P. Yang and J. C. Shafer, *Chem. Commun.*, 2018, **54**, 10578-10581.
